# *catena*-Poly[[copper(II)-bis­[μ-bis­(pyridin-3-yl)methanone-κ^2^ *N*:*N*′]] bis­(tetra­fluorido­borate)]

**DOI:** 10.1107/S1600536811050628

**Published:** 2011-11-30

**Authors:** Bin Liu

**Affiliations:** a Department of Chemistry, Capital Normal University, Beijing 100048, People’s Republic of China

## Abstract

In the title complex, {[Cu(C_11_H_8_N_2_O)_2_](BF_4_)_2_}_*n*_, the Cu^II^ ion is situated on an inversion centre and adopts an N_4_F_2_ octa­hedral coordination geometry with four N atoms from four different bis­(pyridin-3-yl)methanone ligands at the equatorial sites and two independent tetra­fluoridoborate anions weakly bonded at the axial sites *via* two F atoms [Cu⋯F = 2.613 (3) Å]. Chains with the bridging ligands are formed along the *a* axis. C—H⋯F inter­actions stabilize the structure. C—O⋯π inter­actions also occur.

## Related literature

For background to coordination chemistry based on pyridyl­methanone derivatives, see: Dendrinou-Samara *et al.* (2003 ▶); Boudalis *et al.* (2003 ▶). For transition metal complexes of di-3-pyridinyl­methanone, see: Chen *et al.* (2005 ▶); Chen & Mak (2005 ▶); Chen *et al.* (2009 ▶). For a comparable structure, see: Wan *et al.* (2008 ▶). 
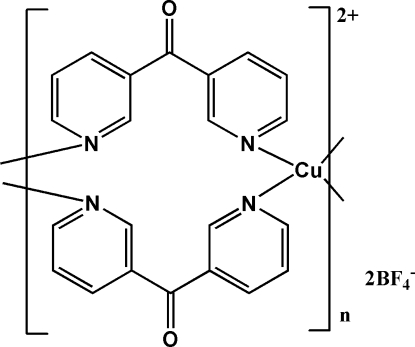


## Experimental

### 

#### Crystal data

[Cu(C_11_H_8_N_2_O)_2_](BF_4_)_2_
*M* *_r_* = 605.55
Triclinic, 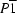

*a* = 7.5542 (13) Å
*b* = 8.7861 (15) Å
*c* = 10.3389 (17) Å
α = 101.280 (2)°
β = 109.236 (2)°
γ = 108.869 (2)°
*V* = 576.96 (17) Å^3^
*Z* = 1
Mo *K*α radiation
μ = 1.04 mm^−1^
*T* = 296 K
0.31 × 0.20 × 0.12 mm

#### Data collection

Bruker APEXII CCD area-detector diffractometer
Absorption correction: multi-scan (*SADABS*; Bruker, 2007 ▶) *T*_min_ = 0.756, *T*_max_ = 1.000
4090 measured reflections
2857 independent reflections
2638 reflections with *I* > 2σ(*I*)
*R*_int_ = 0.023

#### Refinement

*R*[*F* ^2^ > 2σ(*F* ^2^)] = 0.049
*wR*(*F* ^2^) = 0.135
*S* = 1.05
2857 reflections
178 parameters
H-atom parameters constrained
Δρ_max_ = 0.85 e Å^−3^
Δρ_min_ = −0.67 e Å^−3^

### 

Data collection: *APEX2* (Bruker, 2007 ▶); cell refinement: *SAINT* (Bruker, 2007 ▶); data reduction: *SAINT*; program(s) used to solve structure: *SHELXS97* (Sheldrick, 2008 ▶); program(s) used to refine structure: *SHELXL97* (Sheldrick, 2008 ▶); molecular graphics: *SHELXTL* (Sheldrick, 2008 ▶); software used to prepare material for publication: *SHELXTL* and *PLATON* (Spek, 2009 ▶).

## Supplementary Material

Crystal structure: contains datablock(s) I, global. DOI: 10.1107/S1600536811050628/bt5715sup1.cif

Structure factors: contains datablock(s) I. DOI: 10.1107/S1600536811050628/bt5715Isup2.hkl

Additional supplementary materials:  crystallographic information; 3D view; checkCIF report

## Figures and Tables

**Table 1:** Selected bond lengths (Å)

Cu1—N1	2.017 (2)
Cu1—N2^i^	2.039 (2)

Symmetry codes: (i) 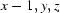.

**Table 2:** Hydrogen-bond geometry (Å, °)

*D*—H⋯*A*	*D*—H	H⋯*A*	*D*⋯*A*	*D*—H⋯*A*
C2—H2*A*⋯F2^ii^	0.93	2.32	3.182 (3)	154
C10—H10*A*⋯F4^iii^	0.93	2.41	3.228 (2)	147

Symmetry codes: (ii) 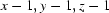; (iii) 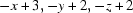.

**Table 3:** C=O⋯π-electron ring inter­actions (Å, °) *Cg*1 and *Cg*2, are the centroids of the N1/C1–C5 and N2/C7–C11 rings, respectively.

C=O⋯*Cg*	O⋯*Cg*	C⋯*Cg*	C=O⋯*Cg*
C6=O1⋯*Cg*1^iv^	3.123 (4)	4.019 (3)	130.79 (2)
C6=O1⋯*Cg*2^v^	3.237 (3)	4.123 (2)	130.20 (1)

Symmetry codes: (iv) 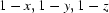; (v) 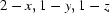.

**Fig. 1.**
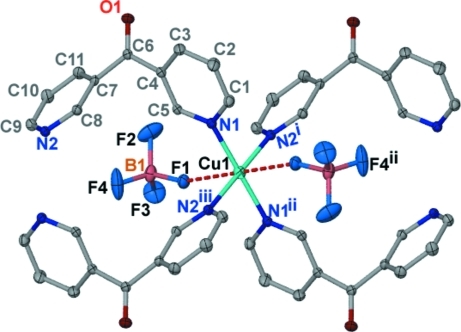
The title complex showing the atom-numbering scheme, with displacement ellipsoids shown at the 30% probability level. Hydrogen atoms are shown as spheres of arbitrary radius. Symmetry codes: (i) x–1, y, z;(ii)–x+1, –y+1, –z+2; (iii)–x+2, –y+1, –z+2.

**Fig. 2.**
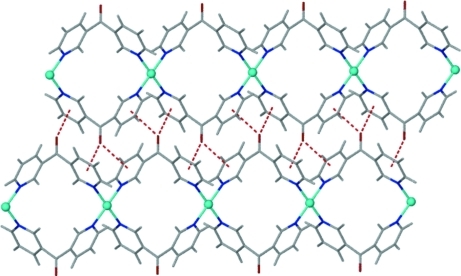
The C═O···π interaction between the infinite cationic chain structure along the a axial direction. The BF4- anions are omitted for clarity.

**Fig. 3.**
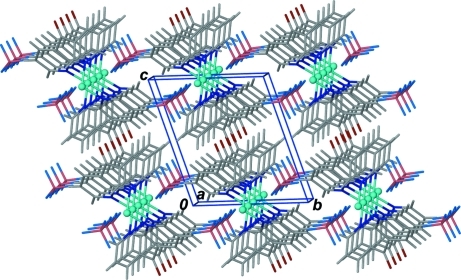
The packing structure of the title complex viewed the a direction.

## supplementary crystallographic information

### Comment

The transition metal complexes of di-2-pyridinylmethanone (di-2-pyridyl ketone) have been widely studied in the passed decade (Dendrinou-Samara *et al.*, 2003; Boudalis *et al.*, 2003). The positional isomer di-3-pyridinylmethanone (di-3-pyridyl ketone) was mostly used as a flexible linker in construction various coordination frameworks. The angular C(*sp*^2^)-CO-C(*sp*^2^) moiety and the rotatable C-C σ bond exhibit subtile tuning on the ligand conformation and subsequent on the formation of various coordination frameworks, such as one-dimensional helical and zigzag  chains (Chen & Mak, 2005), two-dimensional nets (Chen *et al.*, 2005) as well as honeycomb-like three-dimensional frameworks (Chen *et al.*, 2009) were constructed. Here we report a new structure derived from di-3-pyridinylmethanone, namely poly{[µ_2_-(bis(3-pyridyl)methanone-κ^2^*N*:*N'*)] copper(II)}ditetrafluoridoborate.

In the title complex, C_22_H_16_B_2_CuF_8_N_4_O_2_, the Cu^II^ ion adopts an N4F2-octahedral coordination geometry with four separate di-3-pyridinylmethanone ligands providing four N atoms at the equatorial sites, while two independent tetrafluoridoborate weakly bonding at the axial sites via two F atoms (Fig. 1). The Cu1···F1 distance is 2.613 (2) Å, comparable to that 2.677 (3) Å in [(CuL_2_)(BF_4_)_2_]_∞_ (L = di-3-pyridinylmethanone, Chen *et al.* 2005), wherein the Cu^II^ adopts a similar N4F2-octahedral geometry. Along the *a* axis, one double bridged chain with the bidentate bridging ligands is formed in the title complex, which is remarkable different from the (4,4) net in [(CuL_2_)(BF_4_)_2_]_∞_ (L = di-3-pyridinylmethanone, Chen *et al.* 2005). The chains are arranged in a shoulder-to-shoulder mode and interconnected through C═O···π(pyridyl) interaction, forming a layer in the *ac* plane (Fig. 2). For the C═O···π(pyridyl) interaction, each C6═O1 points to the opposite chain and is embraced by two pyridyl rings. The O1···Cg(pyridyl) distances lie within the 3.123 (4)-3.237 (3) Å range (Table 1), well comparable to that 2.916-3.125 Å in Cu(L)_2_(BF_4_)_2_ (L = 2,6-pyridinediylbis(3-pyridinyl)methanone) reported by Wan *et al.* (Wan *et al.* 2008). The formed layers are almost parallel and stacked along the *b* direction to furnish a three-dimensional framework, with the tetrafluoridoborate anions embedded among the interstices (Fig. 3). C—H(pyridyl)···F interactions is also found to stabilize the full framework (Table 1).

### Experimental

Di-3-pyridinylmethanone was prepared according to the previously reported procedure (Chen & Mak 2005). Cu(BF_4_)_2_.xH_2_O (40 mg) and di-3-pyridylmethanone (19mg, 0.1 mmol) were mixed and dissolved in 4 ml acetonitrile with stirring at room temperature. To the solution 1 ml methanol was subsequently dropped, obtaining a clear solution. Filtration was conducted the filtrate was left to evaporate in air. The needle-like crystals were deposited after one week (15.4 mg, 51% yield based on ligand).

### Refinement

All H atoms were located in the difference electron density maps but were placed in idealized positions and allowed to ride on the carrier atoms, with C—H = 0.93 Å and with *U*_iso_(H) = 1.2*U*_eq_(C).

### Crystal data

**Table:**

[Cu(C_11_H_8_N_2_O)_2_](BF_4_)_2_	*Z* = 1
*M**_r_* = 605.55	*F*(000) = 303
Triclinic, *P*1	*D*_x_ = 1.743 Mg m^−^^3^
Hall symbol: -P 1	Mo *K*α radiation, λ = 0.71073 Å
*a* = 7.5542 (13) Å	Cell parameters from 202 reflections
*b* = 8.7861 (15) Å	θ = 2.2–28.6°
*c* = 10.3389 (17) Å	µ = 1.04 mm^−^^1^
α = 101.280 (2)°	*T* = 296 K
β = 109.236 (2)°	Needle, blue
γ = 108.869 (2)°	0.31 × 0.20 × 0.12 mm
*V* = 576.96 (17) Å^3^	

### Data collection

**Table:**

'Bruker ApEXII CCD area-detector' diffractometer	2857 independent reflections
Radiation source: fine-focus sealed tube	2638 reflections with *I* > 2σ(*I*)
graphite	*R*_int_ = 0.023
ω scans	θ_max_ = 28.6°, θ_min_ = 2.2°
Absorption correction: multi-scan (*SADABS*; Bruker, 2007)	*h* = −10→10
*T*_min_ = 0.756, *T*_max_ = 1.000	*k* = −11→6
4090 measured reflections	*l* = −13→13

### Refinement

**Table:**

Refinement on *F*^2^	Primary atom site location: structure-invariant direct methods
Least-squares matrix: full	Secondary atom site location: difference Fourier map
*R*[*F*^2^ > 2σ(*F*^2^)] = 0.049	Hydrogen site location: inferred from neighbouring sites
*wR*(*F*^2^) = 0.135	H-atom parameters constrained
*S* = 1.05	*w* = 1/[σ^2^(*F*_o_^2^) + (0.076*P*)^2^ + 0.4634*P*] *P* = (*F*_o_^2^ + 2*F*_c_^2^)/3
2857 reflections	(Δ/σ)_max_ < 0.001
178 parameters	Δρ_max_ = 0.85 e Å^−^^3^
0 restraints	Δρ_min_ = −0.67 e Å^−^^3^

### Special details

**Table:**

Geometry. All esds (except the esd in the dihedral angle between two l.s. planes) are estimated using the full covariance matrix. The cell esds are taken into account individually in the estimation of esds in distances, angles and torsion angles; correlations between esds in cell parameters are only used when they are defined by crystal symmetry. An approximate (isotropic) treatment of cell esds is used for estimating esds involving l.s. planes.
Refinement. Refinement of F^2^ against ALL reflections. The weighted R-factor wR and goodness of fit S are based on F^2^, conventional R-factors R are based on F, with F set to zero for negative F^2^. The threshold expression of F^2^ > 2sigma(F^2^) is used only for calculating R-factors(gt) etc. and is not relevant to the choice of reflections for refinement. R-factors based on F^2^ are statistically about twice as large as those based on F, and R- factors based on ALL data will be even larger.

### Fractional atomic coordinates and isotropic or equivalent isotropic displacement parameters (Å^2^)

**Table:**

	*x*	*y*	*z*	*U*_iso_*/*U*_eq_	
Cu1	0.5000	0.5000	1.0000	0.02531 (15)	
O1	0.7976 (3)	0.5511 (3)	0.4912 (2)	0.0449 (5)	
N1	0.5354 (3)	0.4065 (3)	0.8203 (2)	0.0275 (4)	
N2	1.3670 (3)	0.6359 (3)	0.8959 (2)	0.0282 (4)	
C1	0.3910 (4)	0.2547 (3)	0.7229 (3)	0.0339 (5)	
H1A	0.2892	0.1911	0.7467	0.041*	
C2	0.3863 (5)	0.1881 (4)	0.5887 (3)	0.0403 (6)	
H2A	0.2844	0.0816	0.5242	0.048*	
C3	0.5354 (4)	0.2825 (4)	0.5519 (3)	0.0375 (6)	
H3A	0.5326	0.2430	0.4607	0.045*	
C4	0.6901 (4)	0.4379 (3)	0.6541 (3)	0.0284 (5)	
C5	0.6860 (4)	0.4953 (3)	0.7873 (3)	0.0288 (5)	
H5A	0.7905	0.5985	0.8559	0.035*	
C6	0.8475 (4)	0.5442 (4)	0.6130 (3)	0.0316 (5)	
C7	1.0686 (4)	0.6398 (3)	0.7218 (3)	0.0293 (5)	
C8	1.1621 (4)	0.5673 (3)	0.8150 (3)	0.0287 (5)	
H8A	1.0798	0.4672	0.8220	0.034*	
C9	1.4820 (4)	0.7852 (4)	0.8909 (3)	0.0358 (6)	
H9A	1.6237	0.8348	0.9476	0.043*	
C10	1.3982 (5)	0.8686 (4)	0.8047 (4)	0.0431 (7)	
H10A	1.4818	0.9739	0.8063	0.052*	
C11	1.1897 (5)	0.7938 (4)	0.7166 (3)	0.0389 (6)	
H11A	1.1310	0.8453	0.6548	0.047*	
F1	0.8566 (3)	0.7588 (3)	1.0928 (2)	0.0494 (5)	
F2	1.1230 (5)	0.8509 (4)	1.3095 (3)	0.1019 (11)	
F3	1.1012 (6)	1.0340 (3)	1.1893 (4)	0.0985 (10)	
F4	1.1711 (4)	0.8119 (4)	1.1061 (5)	0.1179 (14)	
B1	1.0679 (5)	0.8698 (5)	1.1734 (5)	0.0480 (8)	

### Atomic displacement parameters (Å^2^)

**Table:**

	*U*^11^	*U*^22^	*U*^33^	*U*^12^	*U*^13^	*U*^23^
Cu1	0.0249 (2)	0.0327 (2)	0.0226 (2)	0.01406 (17)	0.01259 (16)	0.01010 (16)
O1	0.0428 (11)	0.0707 (15)	0.0306 (10)	0.0272 (11)	0.0183 (9)	0.0254 (10)
N1	0.0273 (10)	0.0341 (10)	0.0255 (9)	0.0152 (8)	0.0134 (8)	0.0110 (8)
N2	0.0275 (10)	0.0348 (10)	0.0260 (9)	0.0152 (8)	0.0132 (8)	0.0106 (8)
C1	0.0329 (13)	0.0357 (13)	0.0342 (13)	0.0135 (10)	0.0171 (10)	0.0105 (10)
C2	0.0357 (14)	0.0396 (14)	0.0314 (13)	0.0089 (11)	0.0113 (11)	0.0003 (11)
C3	0.0373 (14)	0.0460 (15)	0.0252 (12)	0.0169 (12)	0.0136 (10)	0.0045 (10)
C4	0.0247 (11)	0.0402 (13)	0.0252 (11)	0.0177 (10)	0.0118 (9)	0.0116 (10)
C5	0.0262 (11)	0.0363 (12)	0.0237 (11)	0.0133 (10)	0.0112 (9)	0.0083 (9)
C6	0.0306 (12)	0.0444 (14)	0.0299 (12)	0.0214 (11)	0.0170 (10)	0.0156 (10)
C7	0.0286 (11)	0.0379 (13)	0.0281 (11)	0.0164 (10)	0.0159 (9)	0.0133 (10)
C8	0.0271 (11)	0.0343 (12)	0.0287 (11)	0.0131 (9)	0.0152 (9)	0.0128 (9)
C9	0.0275 (12)	0.0361 (13)	0.0412 (14)	0.0109 (10)	0.0139 (11)	0.0133 (11)
C10	0.0384 (15)	0.0359 (14)	0.0604 (19)	0.0145 (12)	0.0226 (14)	0.0259 (13)
C11	0.0398 (14)	0.0434 (15)	0.0468 (15)	0.0227 (12)	0.0223 (12)	0.0262 (13)
F1	0.0304 (8)	0.0654 (12)	0.0413 (10)	0.0127 (8)	0.0117 (7)	0.0142 (9)
F2	0.101 (2)	0.0750 (17)	0.0580 (15)	0.0260 (16)	−0.0246 (15)	−0.0022 (13)
F3	0.116 (3)	0.0466 (13)	0.127 (3)	0.0285 (15)	0.053 (2)	0.0236 (15)
F4	0.0569 (16)	0.099 (2)	0.167 (3)	0.0172 (15)	0.064 (2)	−0.018 (2)
B1	0.0310 (15)	0.0391 (17)	0.057 (2)	0.0095 (13)	0.0122 (14)	0.0016 (15)

### Geometric parameters (Å, °)

**Table:**

Cu1—N1^i^	2.017 (2)	C4—C5	1.385 (3)
Cu1—N1	2.017 (2)	C4—C6	1.496 (3)
Cu1—N2^ii^	2.039 (2)	C5—H5A	0.9300
Cu1—N2^iii^	2.039 (2)	C6—C7	1.498 (4)
O1—C6	1.210 (3)	C7—C8	1.384 (4)
N1—C1	1.339 (3)	C7—C11	1.390 (4)
N1—C5	1.344 (3)	C8—H8A	0.9300
N2—C9	1.341 (3)	C9—C10	1.384 (4)
N2—C8	1.344 (3)	C9—H9A	0.9300
N2—Cu1^iv^	2.039 (2)	C10—C11	1.376 (4)
C1—C2	1.380 (4)	C10—H10A	0.9300
C1—H1A	0.9300	C11—H11A	0.9300
C2—C3	1.379 (4)	F1—B1	1.411 (4)
C2—H2A	0.9300	F2—B1	1.390 (5)
C3—C4	1.391 (4)	F3—B1	1.348 (5)
C3—H3A	0.9300	F4—B1	1.352 (5)
			
N1^i^—Cu1—N1	180.000 (1)	C4—C5—H5A	119.0
N1^i^—Cu1—N2^ii^	91.68 (8)	O1—C6—C4	120.2 (2)
N1—Cu1—N2^ii^	88.32 (8)	O1—C6—C7	119.8 (2)
N1^i^—Cu1—N2^iii^	88.32 (8)	C4—C6—C7	120.0 (2)
N1—Cu1—N2^iii^	91.68 (8)	C8—C7—C11	118.9 (2)
N2^ii^—Cu1—N2^iii^	180.000 (1)	C8—C7—C6	121.1 (2)
C1—N1—C5	118.2 (2)	C11—C7—C6	119.5 (2)
C1—N1—Cu1	118.20 (17)	N2—C8—C7	122.6 (2)
C5—N1—Cu1	123.27 (17)	N2—C8—H8A	118.7
C9—N2—C8	117.9 (2)	C7—C8—H8A	118.7
C9—N2—Cu1^iv^	121.30 (18)	N2—C9—C10	122.7 (3)
C8—N2—Cu1^iv^	120.26 (17)	N2—C9—H9A	118.7
N1—C1—C2	123.0 (2)	C10—C9—H9A	118.7
N1—C1—H1A	118.5	C11—C10—C9	119.2 (3)
C2—C1—H1A	118.5	C11—C10—H10A	120.4
C3—C2—C1	118.8 (3)	C9—C10—H10A	120.4
C3—C2—H2A	120.6	C10—C11—C7	118.6 (3)
C1—C2—H2A	120.6	C10—C11—H11A	120.7
C2—C3—C4	118.7 (2)	C7—C11—H11A	120.7
C2—C3—H3A	120.7	F3—B1—F4	114.2 (4)
C4—C3—H3A	120.7	F3—B1—F2	109.2 (3)
C5—C4—C3	119.1 (2)	F4—B1—F2	108.9 (4)
C5—C4—C6	121.7 (2)	F3—B1—F1	111.6 (3)
C3—C4—C6	119.0 (2)	F4—B1—F1	106.8 (3)
N1—C5—C4	122.0 (2)	F2—B1—F1	105.8 (3)
N1—C5—H5A	119.0		
			
N1^i^—Cu1—N1—C1	−85 (100)	C3—C4—C6—O1	37.1 (4)
N2^ii^—Cu1—N1—C1	−95.5 (2)	C5—C4—C6—C7	42.9 (4)
N2^iii^—Cu1—N1—C1	84.5 (2)	C3—C4—C6—C7	−141.6 (3)
N1^i^—Cu1—N1—C5	89 (100)	O1—C6—C7—C8	−138.8 (3)
N2^ii^—Cu1—N1—C5	78.4 (2)	C4—C6—C7—C8	39.9 (3)
N2^iii^—Cu1—N1—C5	−101.6 (2)	O1—C6—C7—C11	32.6 (4)
C5—N1—C1—C2	−2.1 (4)	C4—C6—C7—C11	−148.6 (3)
Cu1—N1—C1—C2	172.0 (2)	C9—N2—C8—C7	3.7 (4)
N1—C1—C2—C3	−0.7 (5)	Cu1^iv^—N2—C8—C7	−168.10 (19)
C1—C2—C3—C4	2.8 (5)	C11—C7—C8—N2	−3.2 (4)
C2—C3—C4—C5	−2.0 (4)	C6—C7—C8—N2	168.3 (2)
C2—C3—C4—C6	−177.6 (3)	C8—N2—C9—C10	−1.0 (4)
C1—N1—C5—C4	2.9 (4)	Cu1^iv^—N2—C9—C10	170.7 (2)
Cu1—N1—C5—C4	−170.93 (18)	N2—C9—C10—C11	−2.2 (5)
C3—C4—C5—N1	−0.9 (4)	C9—C10—C11—C7	2.7 (5)
C6—C4—C5—N1	174.6 (2)	C8—C7—C11—C10	−0.1 (4)
C5—C4—C6—O1	−138.3 (3)	C6—C7—C11—C10	−171.7 (3)

Symmetry codes: (i) −*x*+1, −*y*+1, −*z*+2; (ii) *x*−1, *y*, *z*; (iii) −*x*+2, −*y*+1, −*z*+2; (iv) *x*+1, *y*, *z*.

### Hydrogen-bond geometry (Å, °)

**Table:**

*D*—H···*A*	*D*—H	H···*A*	*D*···*A*	*D*—H···*A*
C2—H2A···F2^v^	0.93	2.32	3.182 (3)	154
C10—H10A···F4^vi^	0.93	2.41	3.228 (2)	147

Symmetry codes: (v) *x*−1, *y*−1, *z*−1; (vi) −*x*+3, −*y*+2, −*z*+2.

### Table 3 C═O···π-electron ring interactions (Å, °)

*Cg*1 and *Cg*2, are the centroids of the N1/C1–C5 and N2/C7–C11 rings, respectively.

**Table:**

C═O···*Cg*	O···*Cg*	C···*Cg*	C═O···*Cg*
C6═O1···*Cg*1^iv^	3.123 (4)	4.019 (3)	130.79 (2)
C6═O1···*Cg*2^v^	3.237 (3)	4.123 (2)	130.20 (1)

Symmetry codes: (iv) -x+1, -y+1, -z+1; (v) -x+2, -y+1, -z+1.
